# NADPH Oxidases: Redox Regulators of Stem Cell Fate and Function

**DOI:** 10.3390/antiox10060973

**Published:** 2021-06-17

**Authors:** Tullia Maraldi, Cristina Angeloni, Cecilia Prata, Silvana Hrelia

**Affiliations:** 1 Department of Biomedical, Metabolic and Neural Sciences, University of Modena and Reggio Emilia, Via del Pozzo 71, 41124 Modena, Italy; tullia.maraldi@unimore.it; 2 School of Pharmacy, University of Camerino, Via Gentile III da Varano, 62032 Camerino, Italy; cristina.angeloni@unicam.it; 3 Department of Pharmacy and Biotechnology, Alma Mater Studiorum—University of Bologna, Via Irnerio 48, 40126 Bologna, Italy; 4 Department for Life Quality Studies, Alma Mater Studiorum—University of Bologna, Corso d’Augusto 237, 47921 Rimini, Italy; silvana.hrelia@unibo.it

**Keywords:** NADPH oxidases, stem cells, reactive oxygen species

## Abstract

One of the major sources of reactive oxygen species (ROS) generated within stem cells is the nicotinamide adenine dinucleotide phosphate (NADPH) oxidase family of enzymes (NOXs), which are critical determinants of the redox state beside antioxidant defense mechanisms. This balance is involved in another one that regulates stem cell fate: indeed, self-renewal, proliferation, and differentiation are decisive steps for stem cells during embryo development, adult tissue renovation, and cell therapy application. Ex vivo culture-expanded stem cells are being investigated for tissue repair and immune modulation, but events such as aging, senescence, and oxidative stress reduce their ex vivo proliferation, which is crucial for their clinical applications. Here, we review the role of NOX-derived ROS in stem cell biology and functions, focusing on positive and negative effects triggered by the activity of different NOX isoforms. We report recent findings on downstream molecular targets of NOX-ROS signaling that can modulate stem cell homeostasis and lineage commitment and discuss the implications in ex vivo expansion and in vivo engraftment, function, and longevity. This review highlights the role of NOX as a pivotal regulator of several stem cell populations, and we conclude that these aspects have important implications in the clinical utility of stem cells, but further studies on the effects of pharmacological modulation of NOX in human stem cells are imperative.

## 1. Introduction

Stem cells are characterized by two key abilities that define stemness: the first is self-renewal, i.e., the capability to maintain a pool of undifferentiated stem cells through symmetric and asymmetric cell divisions; the second is related to the differentiation potential and is evaluated as levels of potency [1]. The level of reactive oxygen species (ROS) within the physiological range modulates several signaling pathways and, as far as stem cells are concerned, an appropriate redox balance can favorite the self-renewal property but a change in ROS concentration can lead to the differentiation process [2]. The modulation of both self-renewal and differentiation is fundamental during embryogenesis and tissue homeostasis throughout life [3]. Interestingly, depending on the differentiation stage, the level of ROS is modified. For example, a ROS increase triggers the proliferation pathway of mesenchymal stem cells (MSCs), while affects the potency of embryonic stem cells (ESCs) and the genomic stability of induced pluripotent stem cells (iPSCs) [2].

The balance between pluripotent embryonic and multipotent adult stem cells is related to metabolism variation that influences the cellular redox state. In turn, ROS can be considered to be signaling molecules that mediate the interplay between metabolism and stem cell fate [3].

Stem cells could be employed in regenerative medicine both in the state of undifferentiated and already differentiated cells to repair tissues and organs where inflammation often takes part in the damage [4]. Cells modulate ROS levels to react to environmental or endogenous *stimuli,* triggering specific signaling pathways, and NADPH oxidase family enzymes (NOXs) represent the main control point for the regulation of the redox state.

From this point of view, it could be intriguing to explore how the redox state is modulated by NOXs to influence metabolism and stem cell fate. In this review, we will describe how NOX function is in general related to the control of stem cell characteristics, and then we will focus on the different stem cell types with the purpose to dissect the eventual application of NOX modulation to enhance their therapeutic potential.

## 2. NOX Structure and Function

The NOX family represents enzymes that produce ROS involved in physiological functions [5]. They are involved in many processes, including host defense, proliferation, regulation of gene expression, wound healing, and cell differentiation during embryogenesis. However, in case of uncontrolled increased activation or downregulation, pathological events occur [6], such as several degenerative and inflammatory diseases and the development and progression of cancer [7].

The NOX family is a group of transmembrane proteins able to transport electrons from NAD(P)H and to oxygen, generating superoxide anion (O_2_^−^), that in some isoforms or by means of superoxide dismutase (SOD) can be transformed into hydrogen peroxide (H_2_O_2_) [8]. Due to the specific subcellular localization of the different NADPH oxidases, ROS production is compartmentalized, leading to modulation of intracellular redox signal cascades [9]. NOXs together with the mitochondrial electron transport chain are considered the main ROS sources in cells, even though other potential sources, such as cytochrome p450, xanthine oxidase (XO), or nitric oxide synthase (NOS), contribute to the redox potential.

In mammals, seven NOX isoforms are expressed, namely, NOX1 to NOX5 and DUOX1 (“dual oxidase 1”) and DUOX2 [10]. NADPH oxidases can be found either within the plasma membrane (NOX1–5 and DUOX1-2) or in other subcellular compartments, i.e., mitochondrial membrane (NOX4), the endoplasmic reticulum (NOX2, NOX4, and NOX5), and nuclear membrane (NOX4 and NOX5). Moreover, NOXs can be found in the specialized membrane microdomains *caveolae* and lipid rafts (NOX1), *invadopodia* (NOX1 and NOX4) and focal adhesions (NOX4) [10,11].

Every NOX family member crosses the membrane by means of six transmembrane helices binding two haem cofactors. The C-terminal domain binds FAD that receives the electrons from NADPH and allows electron transfer to the haem group and further across the membrane to molecular oxygen. Different from other NOX isoforms, DUOX1, DUOX2, and NOX5 possess a calcium-binding regions at their N-terminus. DUOX1 and 2 are characterized by a domain with a structure similar to the active site of peroxidase. NOX1, NOX2, NOX3, and NOX5 produce O_2_^−^ while NOX4, DUOX1, and DUOX2 lead to the generation of H_2_O_2_. To obtain an active NOX complex, NOX1-3 requires the assembly of membrane protein p22phox, cytosolic proteins (p47phox, p67phox, p40phox), or the GTP-binding protein Rac1/2. The main role of these subunits is to connect FAD and NADPH in order to facilitate the transport of electrons. Differently, NOX4 interacts only with p22phox not with other proteins, and therefore, it is considered a constitutively active isoform that is regulated at the level of transcript expression. DUOX1, DUOX2, and NOX5 contain EF-hands (helix–loop–helix motifs) that bind calcium ions for activation and their activation is independent from cytosolic subunits [1]. The mechanism of the activation of NOX family enzymes has been recently described in detail [12,13].

Once the active NOX complex is formed, electrons are transferred from NADPH to FAD, causing its reduction to FADH_2_. FADH_2_, through haem-binding sites, reduces molecular oxygen to superoxide anion, that often undergoes dismutation reactions in which one molecule of O_2_**^−^** donates an electron to another, forming H_2_O_2._ This reaction can be catalyzed by SOD isoforms or occurs spontaneously under low pH conditions. As previously reported, H_2_O_2_, rather than O_2_**^−^**, has been identified as a product of NOX4, DUOX1, and DUOX2 but, for thermodynamic reasons, this cannot originate from haem-catalyzed two-electron reduction [1]. It is noteworthy that NOX4 is able to generate H_2_O_2_ as a function of oxygen concentration throughout a physiological range of pO_2_ values and to respond rapidly to changes in pO_2_ [14].

ROS, including NOX-derived superoxide and hydrogen peroxide, can act both as signal molecules and as detrimental agents according to their concentrations and subcellular localization. As far as the redox biosignaling is concerned, ROS represent a group of readily available and precisely localized modulators of the highly sophisticated signaling network that eventually leads to the differentiation of stem cells.

## 3. NOXs and ROS: Effectors and Modulators of Redox and Metabolic Homeostasis in Stem Cells

NOXs, together with the mitochondria electron-transport chain, are the main intracellular sources of ROS [15]. However, ROS generated in mitochondria are a byproducts of cellular respiration meanwhile NOXs deliberately produce ROS, and this production is tightly regulated by the cell [16]. Three major forms of intracellular ROS exist: superoxide anions (O^−^), hydrogen peroxide (H_2_O_2_), and hydroxyl radicals (OH^−^) [17]. They are highly reactive species able to oxidize the main biological macromolecules such as carbohydrates, lipids, proteins, and nucleic acids [5]. Cells can counteract an excessive production of ROS by a complex antioxidant defense system that consists of molecules and enzymes able to transform ROS into more stable and less reactive species. Reduced glutathione (GSH) is the main intracellular antioxidant beside many different enzymes such as superoxide dismutase, which catalyzes the dismutation of O_2_^−^ into oxygen and H_2_O_2_, catalase that produces H_2_O + O_2_ from H_2_O_2_, thioredoxin reductase, and glutathione reductase that keeps thioredoxin and GSH in their reduced state. Moreover, exogenous antioxidants such as vitamin E, vitamin C, and some phytochemicals contribute to the maintenance of the physiological cell redox state. ROS, originally thought to be just a damaging byproduct of metabolism, have been recently revisited as key players in cell fate signaling. ROS modulate redox signaling by oxidizing specific protein residues. It has been shown that cysteine residues and, to a lesser extent, methionine residues are involved in redox signaling [18] because of the presence of sulfur that makes them more sensitive to oxidation [19]. The oxidized forms of these two amino acids can be easily reduced by the antioxidant defenses, making the reactive residues of methionine and cysteines real redox-dependent molecular switches. The reduction of oxidized methionine is catalyzed by methionine sulfoxide reductase [20], while oxidized cysteine is transformed into cysteine by various proteins including glutaredoxins, thioredoxins, and thioredoxins reductase [21]. When ROS levels rise beyond a threshold value, there is an irreversible hyperoxidation of these residues [19,20], which induces permanent damage to the target proteins. For this reason, a fine control of the cellular redox state is essential to maintain the functionality of the target proteins. Interestingly, crucial proteins modulating stem cell self-renewal and differentiation such as HIF-1a, FoxOs, APE1/Ref-1, Nrf2, ATM, p38, and p53 have been identified as redox-sensitive proteins. Hypoxia-inducible factor 1 (HIF-1) is a transcription factor and a master regulator of the cellular response to hypoxia [22]. HIF-1 is involved in the maintenance of both cell-cycle quiescence and stem cell state, playing a key role in preserving the stem cell pool [23,24]. p38 mitogen-activated protein kinase plays a critical role in cell proliferation and differentiation of stem cells [25,26,27]. p53, called “the guardian of the genome” because of its role in conserving stability by preventing genome mutation, has an important role in the regulation of stem cell self-renewal and homeostasis (reviewed in [28]). The ataxia telangiectasia mutated (ATM) protein kinase, involved in maintaining genomic stability [29,30], also regulates the intracellular production of ROS [31] that are determinant for stem cell self-renewal. Nuclear factor erythroid-2-related factor 2 (Nrf2) is involved in the maintenance of cellular redox homeostasis through the up-regulation of antioxidant enzymes. Different studies suggest that Nrf2 is an effective modulator of stem cell self-renewal and differentiation [32,33,34]. Apurinic/apyrimidinic endonuclease 1 (APE1), also called Redox Factor-1 [1], is a pleiotropic protein that regulates various cellular functions including oxidative stress and has a role in the maintenance of the stem cell pool and differentiation by modulating intracellular redox homeostasis [35,36,37]. The FOXO (Forkhead box O) transcription factors are implicated in several signaling pathways including ROS response, cell proliferation, regulation of programmed cell death, longevity, metabolism, and maintenance of stem cell self-renewal [38,39,40,41].

In stem cells, ROS modulate the redox state in synchrony with metabolism, influencing the balance between self-renewal and differentiation [3]. It has been observed that ROS levels are low in niches where stem cells undertake self-renewal, whereas ROS increased in differentiated stem cells [42,43]. In different types of stem cells, both a reduction and an increase in ROS with respect to baseline levels impair their regenerative potential by reducing their proliferation, differentiation, and self-renewal [44,45,46,47,48]. For this reason, optimal ROS levels are critical for proper stem cell function. A slight increase in basal ROS levels has been observed to induce MSC proliferation and migration due to the activation of ERK 1/2- and Jun-1/2-mediated signal transduction pathways [49,50,51]. ROS produced by NOXs seem to play a key role in the expansion of MSCs; in fact the silencing of NOX1 by siRNA prevents the proliferation of MSC induced by IL-17, confirming the key role of this NOX [44]. Other agents that slightly increase ROS levels are capable of enhancing stem cell proliferation as well. These include platelet-derived growth factor BB that increases the proliferation and migration of MSCs obtained from adipose tissue [45]; hypoxia that induces PSC proliferation through MAPK activation, nuclear factor-*κ*B (NF-*κ*B), and Wnt-mediated signaling [11,48]. The enhancement of NOX activity and the consequent increase in ROS mediated by phosphatidylcholine-specific phospholipase C induces the differentiation of rat MSCs to neuron-like cells, suggesting a key role of NOX in determining stem cell fate [52]. Moreover, the prolonged expression of NOX-2 with a consequent increase in ROS plays a key role in the antibacterial function of ESCs [53].

In conclusion, the maintenance of the correct redox state in stem cells is extremely important because both “reductive stress” and “oxidative stress” significantly alter stem cell homeostasis [54]. In the next paragraphs, the role of NADPH oxidases in modulating the redox state of different populations of stem cells will be analyzed.

## 4. Stem Cells and Progenitor Cells: ROS and NOX Functions

### 4.1. Embryonic Stem Cells (ESCs)

ESCs originate from the embryonic inner cell mass, at the blastocyst stage of development and possess the ability to differentiate into all three germ layers of the embryo [55]. ESCs have a shortened G1 cell cycle phase; thus, they self-renew rapidly thanks to a glycolytic and pentose phosphate metabolism, instead of oxidative phosphorylation [56]. Indeed, an immature mitochondrial morphology and a reduced redox environment are typical of ESCs [57]. The enhancement of glycolysis through hypoxia-mediated HIF activation and the inhibition of oxidative phosphorylation improve proliferation and maintenance of ESCs, while repressing differentiation [58].

The environmental oxygen tension and intracellular ROS level play a prominent role in proliferation of ESCs in addition to their differentiation: notably, the early embryonic developmental stages occur under a low oxygen level, around 2.4% prior to implantation [59]. In the case of ROS increase, only a transient G2/M cell cycle arrest occurs in ESCs cultivated in vitro, suggesting that ESCs possess a scavenging machinery to counteract oxidative stress [60]. However, in response to increasing levels of ROS, the ESC markers of pluripotency, OCT4, Nanog, Tra 1-60, and Sox2 decline, triggering the ESC differentiation towards the mesodermal and endodermal lineages. Notably, this event can be avoided by the use of antioxidants [61]. Furthermore, chronic ROS exposure causes apoptosis in ESCs, suggesting that the ESC antioxidant defenses might be exhausted [60]. On the other hand, the prolonged hypoxic environment, leading to a ROS rise, induces apoptosis as well [62]. According to these results, ESCs cultured under physiological oxygen levels (2%) are considered optimal to maintain their genomic integrity and self-renewal [63]. Therefore, a ROS level decrease is linked to an improved stem cell maintenance, and redox status may directly modify the stem cell fate.

As far as signaling is concerned, SIRT1-mediated inhibition of p53 antioxidant function enhances intracellular ROS in mouse ESCs [64]. Moreover, SIRT1 makes ES cells sensitive to ROS and inhibits p53-mediated suppression of NANOG expression [64]. Evidence also suggests that SIRT1 is an important player in the regulation of ESC mitochondria [65]. In a recent study by Bino et al. [66] conducted in mouse p38α deficient ESCs, NOX2/gp91phox was over-expressed, leading to ROS formation. The increase in superoxide formation was confirmed when NOX2 was silenced by siRNA in p38α-deficient cells. These data suggest the importance of p38α kinase in the regulation of ROS metabolism in embryonic stem cells.

Interestingly, in a study by Kucera et al. [67], a low expression of NOXs was reported in a model of mouse embryonic stem cells. However, NOX inhibitors, such as apocynin and diphenyleneiodonium (DPI), impaired proliferation of cells through a prooxidant activity, a kind of side effect of these drugs. More precisely, they showed that apocynin inhibits the PI3K/Akt pathway with its downstream transcriptional factor Nanog. On the contrary, apocynin increased activity of canonical Wnt signaling. Opposite to this, DPI enhanced both PI3K/Akt and Erk signaling pathways without affecting Wnt [67]. These data hint that results obtained with these NOX inhibitors in ESCs should be interpreted in the light of unexpected interactions of these molecules with intracellular signaling pathways, rather than with NOX enzymes.

As it is well known, ESCs are pluripotent stem cells that can efficiently generate all embryonic but not extra-embryonic tissues. Nevertheless, a small percentage (0.1–1%) of totipotent-like cells arise spontaneously in ESC cultures, having expanded cell fate potential to differentiate into both embryonic and extraembryonic cells: these rare cells are also called totipotent-like cells [68]. Zhang et al. recently revealed an abnormal redox state characterized by increased ROS level in totipotent-like cells that appeared spontaneously in ESC culture [68]. DPI significantly decreased the overall ROS level and the percentage of totipotent-like cells. Collectively, this study identified cellular redox state as a pivotal factor regulating the cycling of totipotent-like state in ESCs, and that PIAS4, a small ubiquitin-like modifier (SUMO) ligase, may act downstream of ROS signaling to orchestrate the initiation of early embryonic-like program in ESCs. What causes the shift of redox state in ESCs during the initiation of totipotent-like program and the redox signaling pathway that might shape the epigenetic program in ESCs remain to be discovered [68].

NOX levels and activity seem to be quickly regulated during mouse embryonic stem cell differentiation: p67phox subunit expression is higher in 2–3-day-old embryoid bodies compared to those aged 11–12 days [69].

Collectively, in ESCs the expression of NOX might be low as well as ROS and OXPHOS levels, of which the particular isoform that is expressed must still be investigated.

### 4.2. Perinatal Stem Cells

Perinatal stem cells, such as those from fetal membranes (amnion and chorion), derive from chorionic villi, umbilical cord including Wharton’s jelly and from amniotic fluid (AF) [70]. These cells contained in so young tissues were defined all as broadly multipotent stem cells [71]. The human amniotic membrane (hAM) consists of an epithelial layer, formed by a monolayer of human amniotic epithelial cells (hAECs), and a collagen-rich mesenchymal layer, in which the human amniotic mesenchymal stromal cells (hAMSCs) are embedded [72]. Cells of the hAM can differentiate into cells of all three germ layers in vitro and in vivo [73]. Perinatal stem cells in general possess embryonic stem cell-like differentiation capability and adult stem cell-like immunomodulatory properties [74]. In vivo, cells of the hAM are exposed to low oxygen tension (1–4%) [75]. Hypoxia is a signal driving placental development, and molecular mechanisms linked to cellular adaptations to low oxygen concentration are integral to trophoblast cell differentiation and placentation. During the normal course of pregnancy, oxygen concentrations within the uterus change dramatically [76]. Low oxygen is typical of early pregnancy, whereas higher intrauterine oxygen tensions occur following establishment of the hemochorial placenta. Placental oxygen levels during the first 10 weeks of human pregnancy are reported to be approximately 1–2% O_2_, but increase to about 8% O_2_ during the second trimester of gestation [76]. In general, oxygen tensions present in utero during early gestation activate HIF signaling and promote trophoblast cell expansion [77].

Similarly, oxygen tension in AF stem cells in vivo is around 1.3%, revealing that these cells live in a low oxygen tension environment [78]. Moreover, the expression of pluripotency genes and the proliferation rate are inversely correlated with the content of ROS. Indeed, low oxygen (1%) extends stemness and proliferative features and delays the induction of senescence-associated markers. Hypoxic hAFSCs activate a metabolic shift and increase resistance to pro-apoptotic *stimuli* [79].

Regarding NOX, in another study by Maraldi and coworkers [80], it was demonstrated that NOX4 expression, in particular the nuclear localization (nNOX4), depends on the cell donor and correlates with the expression of transcription factors involved in stemness regulation, such as Oct4, SSEA-4, and Sox2. Furthermore, nNOX4 is linked with the nuclear localization of redox sensitive transcription factors, as Nrf2 and NF-*κ*B, and with the differentiation potential. Taken together, nNOX4 regulation may have important effects in stem cell capability through modulation of transcription factors and DNA damage, since NOX4 can localize into PML nuclear bodies (PML-NB), where it associates with prelamin A. Besides, NOX4 post translational modification, involved in PML-NB localization, is linked to the modulation of the premature aging phenotype occurrence [80].

Together, these data demonstrate that low oxygen concentrations in the perinatal environment should be maintained to improve the generation of functional stem cells for therapeutic use by delaying the onset of cellular aging.

### 4.3. Induced Pluripotent Stem Cells

Induced pluripotency occurs when a cell is reprogrammed to revert to a pluripotent state and becomes what is called an induced pluripotent stem cell (iPSC) [81]. IPSCs combine the advantages of adult and embryonic stem cells. These cells combine pluripotency with the proliferation potential; thus, they are used as a good model for studying diseases as well as drug testing without any ethical concerns. Moreover, these cells can be generated to be patient-specific and/or disease-specific, unlike ESCs [82].

One of the methods used to generate tissue-specific pluripotent cells is via transfection with the transcription factors, Oct4, SOX2, KLF4, and c-MYC (collectively known as the four factors or 4F). Unfortunately, reprogramming adult cells into iPSCs is associated with the increased events of new genomic abnormalities [83]. Checking the integrity of the chromosomes, as well as the genome, is fundamental for approving the safety of newly generated iPSCs in the case of clinical use [84].

During reprogramming of PSCs, mitochondria become progressively smaller and less active; therefore, the cellular metabolism shifts from oxidative respiration to oxidative glycolysis: this process prevents the accumulation of ROS and thus oxidative stress in the cells [85]. Notably, these cells employ autophagy, by which dysfunctional mitochondria and the resulting ROS rise can be rapidly removed, protecting their genome from oxidative damage and thus maintaining their self-renewal and pluripotency [86]. Interestingly, low O_2_ tension is the better condition to obtain efficiency of reprogramming and maintenance of iPSCs [87]. Generally speaking, an increase in ROS levels can result in the modification of nucleotide bases, single and double-strand breaks, as well as telomere attrition. However, Zhou et al. [88] showed that early generation of ROS is required for nuclear reprogramming of somatic cells to pluripotency: moreover, genetic knockdown and knockout of the oxidative enzyme NOX (1–4) or addition of antioxidants suppresses reprogramming. The findings shed light on the ROS-involved mechanisms by which pluripotent stem cells are generated. However, excessive ROS generation using genetic and pharmacological approaches also impaired reprogramming.

Overall, these data suggest that redox signaling is activated early with reprogramming, and optimal levels of ROS signaling, including NOX-derived ones, are essential to induce pluripotency.

### 4.4. Adult Stem Cells

Adult stem cells (ASCs) are multipotent cells that can be found in adult tissues. These cells are characterized by having the ability of self-renewal, as well as differentiation into most of the cell types in the body. ASCs can be found in almost all tissues in the body although in different quantities: they can be easily obtained from bone marrow, dental pulp tissue, skin, and the gastrointestinal tract, but can be isolated with relative ease from adipose tissue, skeletal muscle, and bones [89]. After birth, adult stem cells, residing in a particular area called the niche, continue to guarantee physiological cell turnover but also to recover damaged tissue [3]. However, the potency of adult stem cells is limited to a subset of lineages, and, unlike ESCs, adult stem cells are mainly highly quiescent, a property that is crucial for their self-renewal capacity [89]. Despite their quiescence, adult stem cells can quickly shift their program and start to highly proliferate to regenerate tissue in response to damage or loss. This fine balance between the maintenance of the stem cell pool and the differentiation towards the downstream lineages requires metabolic plasticity that regulates quiescence and a proliferative state [90].

Analyses of hematopoietic stem cells (HSCs), neural stem cells (NSCs), and mesenchymal stem cells (MSCs) revealed a preference for aerobic glycolysis and repression of oxidative phosphorylation [3]. This preference may be due to multiple factors, starting from the low energy requirements during quiescence, and then to the need to protect stem cells from oxidative stress, both of them were obtained by lowering mitochondrial activity and by the localization within a hypoxic niche [91].

On the other hand, Yoneiama et al. in 2010 clearly demonstrated that endogenous ROS and nitric oxide are essential for the proliferation of embryonic neural stem/progenitor cells. In particular, in neural stem cells (NSCs) the stimulation of NOX4-derived superoxide production by angiotensin II significantly increases their proliferation [44,92]; however, the maintenance of neural stem cells is regulated by FOXO3 redox balance and transcriptional control of metabolic genes [93]. Results obtained by Mazzonetto et al. [94] demonstrate that ROS acts as a secondary messenger in the control of cerebellar neural stem/progenitor cell proliferation, and that NOX3-inhibition reverses the excessive proliferation phenotype, indicating that this isoform is involved in the increased proliferation rate observed in granule cell precursors.

ROS generated by NADPH oxidases are also important for the self-renewal of spermatogonial stem cells (SSCs). As reported by Morimoto et al., a connection between NOX3 and the self-renewal potential of mouse SSCs was observed, suggesting that this mechanism is in fact regulated by sequential activation of different NOX genes and may or may not occur through the PIK3-Akt and MAP2K1 pathways [95]. However, the same group recently demonstrated that ROS produced by NADPH1 oxidase 1 (NOX1) drive SSC self-renewal through feed-forward ROS production [96]. Moreover, NOX1-derived ROS also modulated the hypoxic response in vivo because NOX1-deficient undifferentiated spermatogonia expressed reduced levels of HIF1A, a master transcription factor for the hypoxic response. Conversely, suppression of mitochondria-derived ROS did not influence SSC fate, indicating that NOX1-derived ROS are more decisive in SSCs than mitochondria-derived ROS [96].

Moreover, very recently, Liu and co-workers highlighted the involvement of NOX4 in the modulation of Spermatogonial Stem/Progenitor Cells survival mediated by Histone Methyltransferase SETDB1 [97].

Together, these results underscore the importance of the ROS origin and oxygen tension in SSC self-renewal.

Mesenchymal stem cells (MSCs) remain quiescent at the basal level of the ROS condition, while ROS levels increase before the cells enter the S phase of the cell cycle, and antioxidants block the G1-S transition [98]. Similar to the results obtained in AFSCs [80], in adult mesenchymal stem cells, the suppression of NOX using apocynin reverses the aging process, since p53 is reduced, and enhances osteogenic potential [99]. Consistently, increased expression of NOX2 and NOX4 has been reported to accelerate senescence of Ang II-stimulated endothelial progenitor cells [100]. Therefore, ROS play a role even in MSC proliferation; however, elevated ROS in MSCs reduce their engraftment potential and induce apoptosis after transplantation [101]. Urao et al. [102] found that deletion of NOX2 caused reduced stem cell mobilization from the bone marrow to peripheral blood. Similarly, in adipose-derived stem cells (ASCs), NOX4 silencing led to reduced proliferation and cell migration, as well as decreased expression of Oct4 and Rex1 and a lower phosphorylation of Akt, PDGFβ, and ERK1/2 [103].

Collectively, ROS generated by NOX can act as an enhancer of stem cell signaling, leading to a proliferation, but, in the case of ROS excess, it can cause molecule damage: further studies are required to better define the optimal ROS level able to function as secondary messengers in the proliferation pathway and, more precisely, the localization of the NOX isoforms involved.

### 4.5. NOX and Differentiation

Differentiation potential, as well as stem cell potency, is modulated by ROS through a cell signaling effect. For example, ROS can enhance the differentiation of stem cells into cardiomyocytes, endothelial cells, adipocytes, keratinocytes, and neurons [2]. Notably, this is not true for all the tissues: indeed, ROS inhibit osteogenesis but enhance the differentiation of cartilage to the hypertrophic stage, leading to chondrocyte death and cartilage degeneration [101]. Thus, the ROS level, depending on the environment but also the intrinsic activity of the cells, is crucial in the regulation of stem cell differentiation in the body. The role of NOX in this process seems to be critical.

In particular, NOX4 has been reported to regulate myogenesis in the myogenic C2C12 cell line, NOX4 expression level correlates with the changes in the presence of the differentiation markers myogenin, i.e., Pax7, MyoD1, and MYyf5. This observation is linked to the changes in MAPK signaling pathways, since modulation of the NOX4 level caused reduction of ERK1/2 phosphorylation during the differentiation [104].

Buggish et al. [105] in 2007 were the first to show that ROS play a crucial role in the differentiation of mouse ES cells toward the cardiovascular cell lineage. During the differentiation, ES cells robustly generate ROS, in particular H_2_O_2_ signaling induced by NOX4 upregulation [106] direct cardiac, and vascular commitment. Indeed, differentiating ES cell expression of NOX1, NOX2, and NOX4 has been demonstrated [107]. Moreover mechanical strain application to embryoid bodies grown from ES cells initiates the cardiovascular differentiation program since a burst of ROS generation occurs, which is followed by the induction of NOX1 and NOX4 and a feed-forward upregulation of ROS production [107]. ROS-mediated signaling cascades in neonatal and ES-cell-derived cardiac cells point towards an involvement of NADPH oxidase in cardiovascular differentiation of ES cells.

Regarding cardiac differentiation of pluripotent stem cells (PSCs), the intracellular ROS and redox balance are carefully regulated by several systems of ROS generation and scavenging, among which NOXs and mitochondria are major sources of intracellular ROS [108].

In addition, NOXs are involved in the differentiation of cardiac cells into cardiac muscle, endothelial, and smooth muscle cells. After silencing NOX2 and NOX4 genes, cardiac precursor cells (CPCs) showed increased levels of the CPC stemness markers c-kit and FIk1 (receptor for vascular endothelial growth factor), while cells overexpressing NOX2 and NOX4 presented a decreased expression of c-kit [109]. These variations were accompanied by fluctuations in the level of Gata4, Gata6, and cytokine-transforming growth factor B1 required for cardiac lineage specification, as well as an altered level of the markers of differentiation, i.e., cardiac troponin T, and α-smooth muscle actin [109]. NOX4 has also been described as a positive driver of the differentiation of mouse embryonic stem cells into smooth muscle cells (SMCs) through the expression of transcription factors essential for the differentiation, namely, serum response factor and myocardin. Moreover, the generation of H_2_O_2_ due to NOX4 activation induced by TGF-β1 drives the differentiation (and maintenance of phenotype) of functional SMC from ESC [110].

Arterial endothelial cell differentiation of mouse induced-pluripotent stem cells (miPSCs) is regulated by NOX2 via the Notch signaling pathway [111]. The expression of EphrinB2, neuropilin 1 (Nrp1), that are example of arterial endothelial markers, and activin receptor-like kinase 1, together with the expression of Notch-pathway components, were significantly decreased in NOX2^−/−^ miPSCs. Consistently, NOX2 upregulation induced a significant increase either of arterial endothelial markers or Notch1 expression, and the same effect was obtained through an activation of Notch. DPI-dependent reduction of ROS or Notch1 silencing blocked this effect in both cases [111]. Remarkably, NOX2 deficiency has been shown to significantly lower many important cell function, such as the potency of vascular repair in mouse ischemic limbs, tube formation, cell migration, cell proliferation, and uptake of Ac-LDL (acetylated low-density lipoprotein) but, notably, to increase sensitivity to oxidative stress [111].

On the other hand, Song et al. in 2014 demonstrated that during vascular differentiation of hESC-derived CD34(+), levels of ROS are primarily generated through NOX4 [112].

Furthermore, differentiation of MSCs towards adipocytes has also been shown to employ NOX4-mediated H_2_O_2_ signaling [113]; this process occurs for profibrotic cell differentiation from adult renal progenitor cells [114] and in the differentiation of osteoblasts from murine 2T3 preosteoblast cells [115].

The last finding is particularly interesting since osteogenic differentiation is usually blocked by a ROS increase, so there is an inverse correlation between the level of ROS and bone differentiation [2]. However, the NOX-derived ROS must be considered as secondary messengers, instead of oxidative stress sources.

Furthermore, NOX4 activity is essential for BMP-induced neuronal differentiation of neural crest stem cells (NCSCs) [116]. Indeed, the silencing of NOX4 in primary NCSCs causes cell death. As NOX4 is the only NOX expressed in NCSCs at a detectable level, different NOX isoforms from other cells might provide ROS for NCSCs during embryogenesis [116]. For example, NOX3 has been demonstrated to be involved in oligodendrocyte differentiation: Accetta et al. unraveled an elaborate network of ROS-generating enzymes (NOX5 to NOX3) activated by PKC necessary for differentiation of oligodendrocytes [117]. Furthermore, NOX5 silencing down-regulated NOX3 mRNA levels, suggesting that ROS produced by NOX5 up-regulate NOX3 expression [117].

Hematopoietic stem cells are a subpopulation of adult SCs in the bone marrow that differentiate into various types of blood cells, including both the myeloid and lymphoid lineages. An increase in ROS intracellular levels is associated with mammalian blood stem cell differentiation and with the accumulation of their immediate progenitors, in which ROS mediate cell cycle progression [118]. Consistent with this, increased ROS regulated myeloproliferation in Foxo3 mutant mice as animal models of human myeloproliferative disorder [119].

Even in this case, NOX4 is the major NOX enzyme involved in the early stages of hematopoietic differentiation from iPSCs and its activity can be involved in the production, the hematopoietic potential, and the phenotype of iPSC-derived CD34^+^ [120]. The presence of NOX in hematopoietic stem cells can have a functional role as O_2_ sensors and/or as low-level ROS producers to be used as redox messengers for controlling cell growth and differentiation [121]. The same group demonstrated that bone marrow-derived human HSPCs are endowed with a composite panel of constitutively active NOXs and express the cell membrane-localized catalytic subunits of the NOX1, NOX2, and NOX4 isoforms [122]. The coordinated activity of the NOX isoforms in HSPC functions likely serves as a secondary messenger. The pro-oxidant setting, triggered when HSPCs leave the hypoxic bone marrow niche, would enable them to be more responsive to proliferative/differentiative *stimuli*. Moreover, enhanced ROS elicit mitochondrial “differentiation” in a pre-commitment phase needed to match the bioenergetic request in the oncoming proliferation/differentiation process [122].

**Figure 1:**
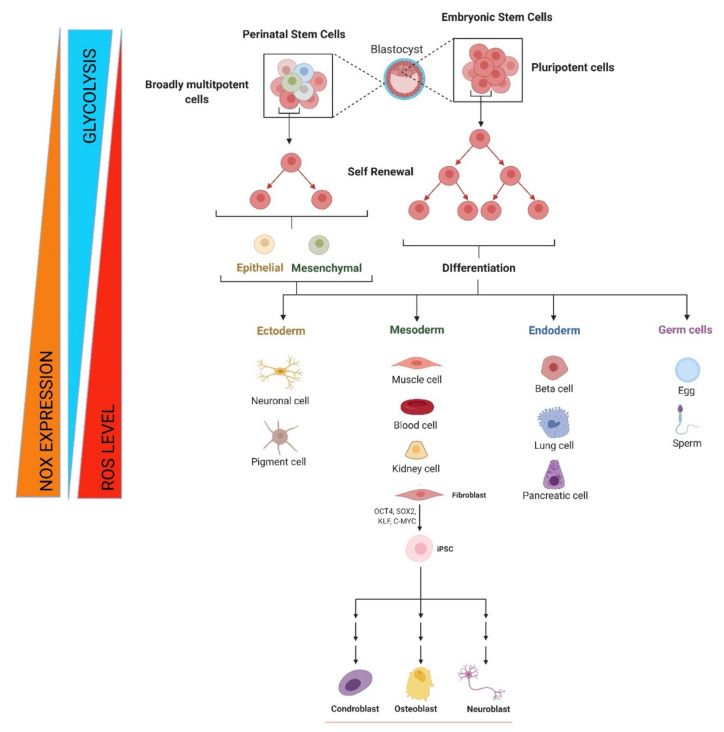
Metabolic and redox changes during stem cell life. Regulation of self-renewal, differentiation, and reprogramming by NOX-derived ROS beside glycolytic metabolism for embryonic, fetal, and adult stem cells. Image created with BioRender.com (accessed on 1 May 2021).

These findings can be interpreted in terms of a positive feed-back mechanism of NOX activation, enabling a fine tuning of the ROS level involved in redox-mediated signaling for growth and differentiation of adult stem cells, as summarized in Figure 1. Additionally, it should be noted that the idea of NOX-mediating differentiation and proliferation is tightly linked to the metabolism regulation; however, NOX role in stem cell senescence and aging has to be taken in consideration during in vitro manipulation.

## 5. NOXs in Hematopoietic Stem Cells: Regulators or Effectors?

Hematopoiesis represents a typical example of cell differentiation: starting from a single cell type, the hematopoietic stem cell (HSC), all mature blood lineages could be obtained. Different from other differentiation processes that are restricted to embryonic development, blood cell production occurs frequently and periodically during the life span. The balance between self-renewal and differentiation in HSCs must be strictly regulated in order to ensure a correct hematopoiesis [123]. Therefore, it is noteworthy to underline the great potential of HSCs in regenerative medicine and the need of a better understanding of HSC biology in order to improve their usefulness.

Several evidence suggests that bone marrow-derived human HSPCs present cell membrane-localized catalytic subunits of the NOX1, NOX2, and NOX4 isoforms and are endowed with a composite panel of constitutively active NOXs [122]. Moreover, a careful modulation of ROS can play a programmatic role in stem cell quiescence and differentiation [124].

Human hematopoietic stem/progenitor cells constitutively generate low levels of hydrogen peroxide whose production is inhibited by apocynin, DPI, catalase, and LY294002 and is scarcely stimulated by PMA (phorbol 12-myristate 13-acetate). Moreover, it is shown that HSCs express at the mRNA and protein levels the catalytic subunits of NOX1, NOX2, and NOX4 isoforms of the NADPH oxidase family along with the complete battery of the regulatory subunits as well as the splicing variant NOX2s. It is noteworthy that the three NOX isoforms are largely co-expressed in the same HSC. These findings suggest a positive feedback mechanism of NOX activation and a potential fine tuning of the ROS level and consequently of redox-mediated signaling involved in HSC growth and differentiation [125]. Moreover, Brault and Colleagues recently reported that NOX4 is the major NOX enzyme involved in the early stages of hematopoietic differentiation from iPSCs and its activity can modulate the production, the hematopoietic potential, and the phenotype of iPSC-derived CD34^+^ [120].

The low level of H_2_O_2_ in quiescent HSCs contributes to maintaining their “stemness”, whereas a higher level of H_2_O_2_ within HSCs or their niche promotes proliferation, differentiation, survival, and migration of HSCs or stem/progenitor cells. In response to ischemic injury, NOX-derived ROS are increased in the bone marrow microenvironment, leading to the expression of hypoxia and hypoxia-inducible factor-1α, that, in turn, helps progenitor cell expansion and mobilization from BM. This process leads to tissue repair and neovascularization.

In pathophysiological states, such as hypertension, atherosclerosis, heart failure, diabetes, and aging, excess amounts of ROS create an inflammatory and oxidative microenvironment, which induces cell damage and apoptosis of the stem and progenitor cells [62].

Although it is certain that a careful regulation of the balance between self-renewal and differentiation of HSCs is critical to ensure the proper function of the blood-forming system [126], much remains to be learned about the role of NOXs as both regulators and effectors in HSCs.

## 6. NOXs and MSC Application: The Fine Tuning among Survival, Proliferation, Differentiation, and Senescence

Several studies report strategies in which NOX expression and activity have been modulated in MSCs in order to obtain better results in their application in stem cell therapy.

Tyurin-Kuzmin and co-workers [127] demonstrated that platelet-derived growth factor (PDGF) orchestrates wound healing and tissue regeneration by regulating recruitment of the precursor MSC and fibroblasts. Actually, PDGF stimulates the generation of H_2_O_2_ derived from NOX4 and DUOX1/2. Indeed, apocynin, cell-permeable catalase and LY294002 prevented PDGF-induced migration and mitotic activity of these cells, suggesting an involvement of H_2_O_2_ and the PI3-kinase pathway. Moreover, silencing of DUOX1/2 in fibroblasts or NOX4 in MSC reduced PDGF-stimulated intracellular H_2_O_2_, Akt phosphorylation, and migration. PDGF-induced migration of mesenchymal cells requires NOX4 and DUOX1/2 enzymes, which mediate redox-sensitive activation of the PI3-kinase-Akt pathway.

Netrin-1 (Ntn-1) is a multifunctional neuronal signaling molecule that improves the tissue-regeneration capacity of stem cells, promoting the proliferation of hUCB-MSCs with regard to the regeneration of injured tissues. The involved mechanism was showed by Lee et al. [128]: Ntn-1 induced the recruitment of NADPH oxidases and Rac1 into membrane lipid rafts to facilitate ROS production via the lipid raft-mediated Inα6β4 signaling pathway. They demonstrated in vivo (models of skin wound healing processes and mouse hindlimb ischemia) that transplantation of hUCB-MSCs pre-treated with Ntn-1 shows a regeneration efficacy through improved vascular regeneration.

Another paracrine factor, such as T-cell-produced IL-17, can induce proliferation of human bone marrow-derived mesenchymal stem cells in a ROS generation-dependent manner. Rac1 GTPase and NOX1 are activated by IL-17 to produce ROS, which in turn stimulates human MSC proliferation, but also induces their migration, motility, and osteoblastic differentiation. Furthermore, IL-17 enhances the expression of M-CSF and receptor activator of NF-kappaB ligand (RANKL) on these stem cells, thereby supporting osteoclastogenesis both in vivo and in vitro [129]. This study confirms what was previously mentioned, so that NOX-derived ROS have to be considered as secondary messengers, instead of oxidative stress sources and can therefore exert a positive effect even in the bone commitment process.

Increasing ROS levels and NADPH oxidase activity can be also a strategy to induce neuronal differentiation in mesenchymal stem cells. The possible mechanism, proposed by Wang et al. [52], may be related to changes in phosphatidylcholine-specific phospholipase C (PC-PLC) activity: D609, an inhibitor of PC-PLC, induced neuronal differentiation in rat MSCs by increasing the ROS level and the activity of NADPH oxidase while the MnSOD and Cu/ZnSOD activities were not altered. Taken together, the results show that PC-PLC mediated neuronal differentiation of rat MSCs by increasing NADPH oxidase activity, ROS level, and up-regulating the Rb protein expression.

However, a negative regulation of NOX can be useful as well: for example, a beneficial effect of MSCs has been proved for intracerebral hemorrhage (ICH), but enhancement of the therapeutic efficacy of MSCs in ICH is necessary. Min et al. [130] recently demonstrated that apocynin treatment, a well-known NADPH oxidase inhibitor, enhances the therapeutic efficacy of MSCs in ICH in the acute stage, such as neuroprotection and the reinforcement of endovascular integrity of cerebral vasculature, through the improvement of the expression of tight junction proteins.

As far as adipose tissues are concerned, oxidative modification occurs during the initial phase of adipogenic differentiation of human adipose tissue-derived stromal cells (hASCs): adipogenic induction in vitro must last a period of seven days in order to stimulate the transition from glycolytic to oxidative energy metabolism. Drehmer et al. [131] showed that ROS production was already increased after three days and may play an important role in the adipogenic differentiation commitment, and NOX represent the main ROS source involved in this process. ROS production did not change after seven days; however, they observed a decrease in the activity of catalase and in non-protein thiol concentration as well as a decreased lipid peroxidation. Thus, a short period of differentiation induction is able to change the energetic and oxidative metabolic profile of hASCs and stimulate cytoprotective processes.

Notably, the in vitro culture of adipose-derived MSC is affected by oxygen tension depending on the cell source, namely, from subcutaneous fat or abdominal adipose tissue. Sela et al. [132] showed that 21% oxygen caused cytostasis of abdominal adipose cells that was accompanied by ROS accumulation and increased expression of NOX1 but not of NOX2 or NOX4. In turn, both 3% oxygen conditions and the exposure to a specific NOX1 inhibitor, ML171, expanded long-term culture, decreased ROS accumulation and apoptosis. This suggests an inhibitory role of NOX1-induced ROS overproduction in abdominal ASCs proliferation, their fat differentiation, and migratory potential. On the contrary, similar cells produced from subcutaneous fat were easily expanded in normoxic cultures, exhibiting low ROS concentrations, a low number of apoptotic cells, and improved fat differentiation and migration.

However, the role of NOX1 and NOX2 activity in mobilization, proliferation, and differentiation of adipocyte progenitors even in visceral white adipose tissue has been proved. In fact, these effects occur in wild-type but not in NOX2 null mice. Thus, NOX2 may provide a therapeutic target to prevent obesity, for example, in the context of sleep disorders such as chronic sleep fragmentation that induces obesity in mice [133]. Furthermore, NOX-induced ROS accumulation and cytokine production by fat are part of the metabolic syndrome.

MSC cultivation in vitro often leads to a senescence process induction of these stem cells [134], so new easy strategies to counteract this event are needed [135]. Since an increase in ROS plays a key role in aging and apoptosis in mesenchymal stem cells derived from bone marrow (BMSCs), blocking NOX could enhance the anti-apoptotic and anti-aging ability of BMSCs counteracting oxidant stress, and thus improving their therapeutic efficacy. Feng et al. [136] showed that the NOX2 inhibitor Acetovanillone and NOX2 siRNA markedly countered the decrease of viability and the increase of aging and apoptosis of BMSCs induced by H_2_O_2_, whereas NOX2 overexpression exacerbated the viability reduction, senescence, and apoptosis of BMSCs. Consistently, the ROS accumulation in BMSCs was also suppressed by NOX2, causing the downregulation of p-p53, p21, p-FoxO1, and Bax and the upregulation of anti-apoptotic protein Bcl-2. In vivo experiments in a model of infarcted myocardium demonstrated higher retention and survival of BMSCs into the host after NOX2 knockdown. The authors concluded that NOX2 inhibition enhances the anti-aging and anti-apoptotic ability of BMSCs and thus promotes survival and retention of BMSCs, which provides a new strategy for improving BMSC-based therapy. In another study by Sun et al. [99] on stem cell aging events, the expression of NOX was suppressed using apocynin, and an enhanced potential for osteogenesis was observed in aging BMSCs. The expression of p53 was shown to be reduced with the suppression of NOX in vitro, and the effect of apocynin in vivo in SAMP6 mice was to increase bone mineral density and total bone volume after three months of treatment. In conclusion, these studies demonstrate that in aging BMSCs, suppression of NADPH oxidase partially reverses the aging process and enhances their therapeutic potential.

MSC cultures are a mix of subpopulations and they are therefore heterogeneous in terms of senescence, differentiation potential, and functional properties. For clinical use, potency assays are useful to monitor cell properties predictive of therapeutic efficacy; on the other hand, the modulation of NOX-derived ROS can be applied to improve this efficacy. However, the therapeutic application and the source of stem cells are crucial to determine if potentiating or blocking NOX could be the successful choice.

## 7. NOX Expression in Neural Stem Cells: Role in the Development and Regenerative Capacity

Neural stem cells (NSCs) are multipotent progenitors that are responsible for generating the three main cell types in the brain, i.e., neurons, astrocytes, and oligodendrocytes, and play a key role in the development and maturation of the central nervous system [92,137]. NSCs are localized in different neurogenic regions during development and in adults. In particular, NSCs are distributed in the ventricular and subventricular zones (SVZ) during development and in the forebrain subventricular zone of the lateral ventricles and the subgranular zone (SGZ) of the dentate gyrus in the hippocampus in adults [138,139]. Interestingly, these neurogenic regions are characterized by high ROS levels [44,140,141]. Tsatmali et al. [141] demonstrated that ROS play a key role in defining the fate of cortical NSCs. Cortical NSCs with high ROS levels differentiate into neurons, while cortical NSCs with lower ROS levels differentiate into astrocytes, oligodendrocytes, and other types of neurons. The role of ROS in cell differentiation was also confirmed by the fact that the use of antioxidants changed the number of neurons that differentiate into the different types of cells. In the last years, several studies have indicated that ROS are mainly produced by NOXs in NCSs in vivo [142] suggesting a critical role of NOX in neuronal differentiation. Suzukawa et al. [143] observed that the use of diphenyleneiodonium (DPI), a NOX-inhibitor, blocked differentiation and neurite out-growth induced by nerve growth factor (NGF) in PC12 cells. The authors demonstrated that the induction of ROS generation by NGF was mediated by Rac1, a well-known activator of the phagocyte NOX [144], suggesting a potential role of a phagocytic-like NOX in neuronal differentiation. Hammed et al. [145] exposed newts to different oxygen tension and confirmed that increased ROS levels are fundamental to stimulate the differentiation of NSCs into neurons. Interestingly, they also observed that this effect was mainly due to NOX activity rather than mitochondria, supporting the knowledge that regulated sources of ROS production have a primary role in modulating signal transduction mediated by ROS. Subsequent studies revealed that NOX4 is the main NOX isoform involved in NSC differentiation. Unlike the other isoforms, NOX4 constitutively produces only minimal amounts of ROS, mainly H_2_O_2_ [5]. The up-regulation of NOX4 represents a mechanism to modulate different cellular processes since H_2_O_2_ produced by NOX4 regulates intracellular signaling [146,147]. Park et al. [16] observed that NOX4 was predominantly expressed among NOX isoforms in SVZ NSCs cultured from mouse neonates and its expression increased during neuronal differentiation. In addition, the treatment of SVZ NSC cultures with the antioxidant N-acetyl cysteine (NAC) reduced neurogenesis as well as the knockdown of NOX4. The involvement of NOX4 in NSC differentiation to neurons was also confirmed by Jiranugrom et al. [148], who investigated hippocampal neurogenesis by measuring different markers of neuronal differentiation in Nox4^−/−^ and WT C57BL/6J mice. Nox4^−/−^ mice had lower doublecortin-positive neuroblasts and dendrites in the SGZ and granular cell layer hippocampal dentate gyrus than WT mice. Moreover, Nox4^−/−^ mice showed higher immature dendrites that had fewer and shorter branches in the middle region of DG than WT mice [148]. Contradictory results have been reported in neural crest stem cells (NCSCs) differentiated with the bone morphogenetic protein 2 (BMP 2) [116]. BMP2 treatment increased the production of ROS, fundamental for NCSC differentiation. However, even if NOX4 was the only detectable NAPDH oxidase, knockdown of this gene induced apoptotic response in NCSCs, making it impossible to dissect detailed functions of ROS or NOX4 in neuronal differentiation. The differentiation of NSCs to astrocytes is modulated by NOX4-produced ROS. Recently, Rodriguez-Vargas et al. [149] demonstrated that Poly (ADP-ribose) polymerase Parp3 controls astrocytic differentiation via NF-kB-regulated NOX4-induced ROS production. In particular, the differentiation of mouse NSCs into astrocytes induced an over-expression of NOX4. The silencing of NOX4 reduced the capacity of the NSCs to differentiate to astrocytes, confirming the crucial role of NOX4 in astroglial differentiation.

NOX enzymes have also been demonstrated to play a pivotal role in the proliferation of NSCs. Embryonic hippocampal neuronal progenitor cells (NPCs) and NSCs exposed to the NOX inhibitor apocynin were not able to form neurospheres, suggesting a crucial role of NOX enzymes in their proliferation [44,92]. The use of DPI reduced ROS production and NSC proliferation induced by 2-[(dimethylamino)methyl]-8-hydroxyquinoline meanwhile neurogenesis was not influenced [150].

Other studies demonstrated the specific role of the different NOX isoforms in NSC proliferation. In vitro, NOX4 overexpression enhanced the proliferation of NSCs and NPSs associated with increased intracellular H_2_O_2_ production and Akt phosphorylation, whereas NOX inhibitors (VAS 2870 and GKT137831) or NOX4 deletion decreased NSC and NPC proliferation triggered by the basic fibroblast growth factor (bFGF) [151]. In vivo, NOX4^−/−^ mice showed a significant reduction of post-injury proliferation of NSCs and neurogenesis in the hippocampus [151]. In the murine neural stem cell line, C17.2, angiotensin II (Ang II), a strong stimulator of NOX, enhanced proliferation and increased superoxide levels [152]. This effect was mediated by NOX4 as Ang II treatment induced NOX4 and siRNA targeting Nox4 mRNA reduced both the constitutive and Ang II-induced NOX4 protein levels and attenuated Ang II-triggered increases in superoxide levels and stem cell proliferation. It has also been observed that, beside NOX4, a high level of ROS produced by NOX2 seems to have a role in physiological NSC proliferation. In particular, Dickinson et al. [153] observed that adult hippocampal stem/progenitor cells (AHPs), a crucial population of cells that proliferate in the brain from development throughout adult life, reacts to growth conditions by increasing NOX2 H_2_O_2_ production. The use of DPI, a relatively non-specific NOX inhibitor, decreased the AHP growth rate in a dose-dependent manner, and the specific role of NOX2 was confirmed using a shRNA construct targeting NOX2. These observations were supported by in vivo data showing that mice lacking functional NOX2 have fewer proliferating NSCs and less adult neurogenesis in the hippocampus. Another study that investigated the role of chemokine (C-X-C motif) ligand 1 (CXCL1) on NCS proliferation demonstrated that CXCL1 administration increased ROS levels and the expression of NOX2/gp91phox in vitro [154]. In addition, the delivery of the NOX inhibitor apocynin into the lateral ventricle of mice blocked CXCL1 from promoting the proliferation of NSCs. Therefore, the authors suggested that CXCL1 promotes ROS production through the NADPH oxidase pathway, which in turn promotes NSC proliferation. If NOX2 is positively correlated with NCS proliferation in the physiological condition, after traumatic brain injury it contributes to oxidative stress and reduces NSC proliferation and neurogenesis [155]. In this context, the use of NOX2-knockout (NOX2-KO) or NOX inhibitor-treated mice during the first week after TBI increased the generation of neuroblasts, enhanced their proliferation, and promoted the maturation and survival of these newly generated NPCs in the perilesional cortex [155].

In conclusion, ROS produced by NOXs play a crucial role in modulating self-renewal and differentiation of NSCs in both development and adult stages. Among NOX isoforms, NOX4 is emerged as crucial for NSC differentiation and proliferation, whereas NOX2 seems to influence only proliferation.

## 8. NOXs in Endothelial Progenitors: Effect on Cell Survival, Proliferation, and Angiogenic Function

Endothelial progenitor cells (EPCs) consist of cells that are able to differentiate into endothelial cells and play a pivotal role in maintaining vascular homeostasis and endothelial integrity and are also importantly involved in the process of neovascularization.

Wang and co-workers highlighted the role of NOX isoforms in angiogenesis, focusing also on EPCs [156]. EPCs display a unique ability to promote angiogenesis and induce endothelial function recovery in injured blood vessels. In this context, Peng and Colleague recently discussed and analyzed studies on the effect of NOX-mediated oxidative stress on the modulation of EPC bioactivities, such as mobilization, migration, and neovascularization, showing that inhibition of NOX improves EPC functions [157].

Therefore, direct or indirect inhibition of NOX isoforms could represent a potential strategy to protect EPCs from oxidative injury and consequently to play a positive role on cell survival, proliferation, and angiogenic functions [158].

Moreover, Li and colleagues [100] studied the NOX homologues that are correlated with late EPC senescence induced by Ang II and the potential inhibitory effect of telmisartan, an angiotensin II receptor antagonist. Results showed the involvement of three different isoforms, even if at different extents: after Ang II stimulation, NOX5 translocation was correlated with early and rapid ROS production, not particularly involved in EPCs senescence. NOX2 and NOX4 contributed greatly to EPC senescence in the late and slow phase. No significant changes in NOX1 or NOX3 were observed. Telmisartan effectively depressed NOX change and delayed late EPC senescence.

It is well known that NOX4 produces H_2_O_2_, acting as a signaling molecule and promoting endothelial cell proliferation and migration as well as protecting against cell death. However, the role of NOX4 in the EPC function is not completely understood. Focusing on this NOX isoform, Hakami and Colleagues isolated EPCs from human saphenous vein and mammary artery discarded during bypass surgery and inhibited NOX4 by means of a Nox4 small interfering RNA (siRNA) (Ad-Nox4i). Results show that NOX4-derived ROS are peculiar for proliferation and migration functions of EPCs and counteract the detrimental effect induced by pro-inflammatory cytokine in EPCs. The authors suggested also that NOX4 could facilitate the efficient function of EPCs, leading to successful neovascularization [159].

NADPH oxidase, thanks to different localization and to the possibility of tuning the amount of ROS generated, represents a peculiar source of ROS for redox signaling related to endothelial cell differentiation. NOX2 is reported to prolong and strengthen intracellular signaling cascades that mediate cytokine-induced signaling, leading to progenitor and early endothelial cells proliferation, instead of their differentiation. In contrast, constitutive activity of NOX4 produces ROS that promote differentiation and stabilization of resulting endothelial cell. ROS in endothelial differentiation represent an extremely sensitive signal modulator. Therefore, any imbalance of ROS formation causes different cellular consequences. In particular, uncontrolled ROS generation and/or ROS production in the incorrect subcellular localization will affect the endothelial differentiaton process. The modulation of ROS production and the subcellular compartmentalization of ROS generation denote a promptly available and highly controlled redox signaling network that, in this case, leads to the differentiation of stem cells into endothelial cells [160].

Moreover, redox signaling by means of NOX activities, controls the maintenance of cell phenotypes and their contribution to intimal thickening and subclinical atherosclerosis. Much remains to be learned about the mechanisms regulating the proliferation, migration, and myogenic differentiation of resident vascular stem cells (vSCs) through NOX-dependent pathways. These details will allow researchers to develop more targeted therapies to counteract subclinical atherosclerosis [161].

In an endothelin-1 (ET-1)–induced apoptosis model of EPCs, pretreatment with ET-1 receptor blocker or NOX inhibitor (apocynin) significantly attenuated the proapoptotic effect of ET-1 on EPCs [162].

In another study, osteoprotegerin, that is considered an independent risk factor for atherosclerotic disease when present at high concentration in serum, was shown to increase apoptosis in EPCs by means of the activation of NOX2 and NOX4 [163].

Another recent study investigated the potential correlation between NOX and EPC functions in hyperlipidemic patients. Although the study involved only a small number of people (30 hyperlipidemic patients and 30 age-matched volunteers), the results showed an increase in NOX expression (NOX2 and NOX4), NOX activity, and consequently ROS production in hyperlipidemic patients, leading to a reduction in EPC functions. This positive correlation between the NOX-mediated oxidative stress and the dysfunctions of circulating EPCs in hyperlipidemic patients suggests that the inhibition and/or downregulation of NOX might be useful as novel strategy to improve EPC functions in hyperlipidemia [164]. The same research group previously obtained similar results in a rat model [165].

Therefore, direct or indirect inhibition of NOX could exert beneficial effects on EPCs, as reported in recent and comprehensive reviews [166].

Since NOX-derived ROS seem to contribute to the dysfunction of EPCs and the development of cardiovascular diseases, NOX might be a potential therapeutic target for counteracting cardiovascular disease.

Interestingly, Medina and Colleagues in a recent consensus document highlighted that accurate cell definitions represent a critical barrier for translation of cell therapies into the clinic [167]. The working definition for EPCs, as cells from circulating blood that promote new blood vessel formation, is not sufficiently accurate in the era of precision medicine. This is especially true as our field progresses toward clinical use of efficacious cell therapy products [168], which require a detailed phenotypic identity, a measurement of purity, and consistent functional readouts as minimal essential release criteria. Therefore, they suggest consideration of endothelial colony-forming cells (ECFC) and myeloid angiogenic cells (MACs) as well-defined cell populations isolated in culture with potential for therapeutic angiogenesis.

## 9. NOX Modulation of Intestinal Stem Cell Proliferation, Differentiation, and Regenerative Function

Intestinal stem cells (ISCs) are essential for maintaining the integrity of the gut epithelium of both mammals and invertebrates and perform essential functions in food digestion and nutrient absorption, and, at the same time, represent a fundamental line of defense against pathogenic bacteria and toxins released into the lumen [169,170,171]. ISCs have the potential to generate differentiated cell types of the intestinal epithelium enterocytes, goblet cells, enteroendocrine cells, and Paneth cells (multipotency) [172].

In mammals, the stem cell compartment is located at the base of the three-dimensional epithelial invaginations forming the crypt niche. The cells originated by the mammalian intestinal stem cells further proliferate before their differentiation into absorptive, mucus secreting, and neuroendocrine epithelial cells [173]. The dynamic renewal of murine epithelia takes 4–5 days [171].

In invertebrates, such as Drosophila, the adult intestine (or midgut) is made up of a single layer of enterocytes in which hormone-producing enteroendocrine cells are also present. Enterocytes of the adult midgut are incessantly replaced by ISCs that are located close to the intestinal basement membrane [174].

Regulation of ISC proliferation is complex. However, there are a few major signaling pathways that are involved, most notably the pathways modulated by Wnt, Epidermal Growth Factor Receptor (EGFR), Hippo, Notch, Hedgehog, and BMP [175]. ROS are central in the regulation of ISC fate as they modulate several of these signaling pathways [176].

The main role of NOXs in the ISC fate seems to be related to their ability to produce ROS in response to a variety of stresses such as commensal bacteria that reside in the gut, toxins, and other environmental factors. Jones et al. [173] investigated the effect of symbiotic *Lactobacilli* on gut epithelial proliferation of both *Drosophila melanogaster* and mice. *D. melanogaster* possesses only two NADPH oxidase: dNOX and dDUOX. The administration of *L. plantarum* induced cell proliferation in the *Drosophila* intestine by a mechanism dependent on the production of ROS by dNOX in enterocytes. Lactate produced by *L. plantarum* induces dNOX activation via a mechanism involving the oxidation of lactate to pyruvate by lactate dehydrogenase, which is accompanied by the transformation of NAD+ to NADH. Then, NADH can be used by the dNOX to generate ROS [177]. Jones et al. [173] confirmed the results obtained in *D. melanogaster* in mammals using wild-type mice and intestinal epithelial cell-specific Nox1-deficient animals. Wild type mice fed with *L. rhamnosus* showed significantly elevated levels of proliferating cells and ROS in the colon compared to controls; this effect was not observed in Nox1-deficient mice, confirming the crucial role of Nox1 in ISC proliferation. Another study carried out in *Drosophila melanogaster* confirmed that dNOX even more than dDUOX is required in enterocytes to activate p38 and promote ISC proliferation in response to pathogenic bacteria [178]. Moreover, unlike DUOX, NOX plays an important role in intestinal regeneration following diverse types of damage such as detergent exposure (SDS) or wounding. An important question remaining is how ROS production by NOX is activated by each stress.

NOX1 plays a crucial role in controlling intestinal stem cells (ISCs) fate. ISCs actively contribute to the regeneration of the colon epithelium, and ISC self-renewal and proliferation are controlled by growth factors (EGFR) and the microbiota. Van der Post et al. [179] demonstrated that ROS produced by NOX1 mediate the proliferation of ISC, in particular colonic cancer cells, thorough the activation of the epidermal growth factor receptor. Interestingly, the microbiota activates Toll-like receptor (TLR) that in turn regulates NOX1 expression, inducing ISC proliferation. Coant et al. [180], using NOX1 knockout mice, demonstrated that NOX1-produced ROS maintain the correct equilibrium between cell proliferation and differentiation in the colon by modulating the pathways mediated by PI3K/Akt, Wnt/β-catenin, and Notch1. In particular, they suggested that NOX1 signaling is critical to maintain the undifferentiated state of the crypt progenitors.

## 10. NOX Regulation of Cancer Stem Cells and Oncogenesis

Many types of cultured cancer cell lines and human tumors at early and late stages of tumorigenesis express higher levels of NOX1, NOX2, NOX4, and NOX5 or their regulatory components compared with normal controls, suggesting a pivotal role either in cancer development or in progression [181,182,183,184]. Cancer stem cells (CSCs) are involved in various tumorigenic process such as invasion, metastasis, angiogenesis, and resistance to chemotherapy [1,185,186]. In contrast to cancer cells, which have elevated ROS levels, CSCs generally maintain low levels of intracellular ROS by means of various mechanism [187,188].

Unfortunately, tumor-initiating cells (TICs)/or cancer stem cells seem to be the most malignant cell subpopulation in tumors because of their resistance to chemotherapy or radiation treatment. Therefore, a potential key innovation for cancer treatment is represented by targeting TICs. In particular, Liu and Colleague [189] showed that PPARγ agonists inhibited the cancer stem cell-like phenotype and decreased tumor growth of human hepatocellular carcinoma (HCC) cells. The increase in NOX2-derived ROS was partially responsible for the inhibitory effects mediated by PPARγ agonists. Nevertheless, ROS generation induced by PPARγ agonist significantly activated Akt, which in turn led to TIC survival by limiting ROS generation. Therefore, much remains to be learned about this topic, and not only NOX2 but also the role played by other isoforms should be consi-dered; this represent a limit of this study, that otherwise suggestss a potential treatment of liver cancer based on a combinatory strategy involving an Akt inhibitor and a PPARγ agonist for inhibition of stem cell-like properties in HCCs.

Focusing now on a different and non-solid tumor, it is difficult to identify a single driving force for leukemogenesis since it is a multistep process. However, the increased production of intracellular ROS characteristics of tumor cells [190] is also a feature of leukemic cells. Elevated intracellular ROS level is indeed a feature observed in numerous leukemic cell lines and also in the cells from patients with different types of leukemia [191,192,193].

As previously reported, a low level of ROS is important for maintaining quiescence and the differentiation potential of hematopoietic stem cells (HSCs), whereas the level of ROS increases during hematopoietic differentiation [194,195]. Analogously, in acute myeloid leukemia (AML), a low level of ROS is associated with leukemic stem cell (LSC) quiescence, whereas a high level promotes blast proliferation [196].

An interesting study, suggesting that cancer stem cells are known to mediate metastasis and recurrence and are therefore a promising therapeutic target, is focused on the CSC inhibitory effect of dihydrotanshinone (DHTS) that involves NOX5 activation. NOX5-derived ROS induced by DHTS deregulated the Stat3/IL-6 pathway, leading to CSC death [197].

Since cell transformation frequently relies on NADPH oxidase-driven ROS production [198,199], NADPH oxidases appear to be suitable therapeutic targets in leukemia as recently reported [200]. In this context, preclinical data show that the inhibition of NADPH oxidases is an effective strategy to block the signaling cascades initiated by the BCR-ABL and FLT3-ITD oncokinases in CML and AML cells, respectively. Thus, the use of TKis and NADPH oxidase inhibitors presents a strong synergistic effect [201]. As discussed above, several oncogenes increase ROS production through NADPH oxidases, which turns these enzymes into desirable targets against leukemia.

Taken together, results suggest distinct and specific signals and effects for NOX family enzymes not only in leukemia but also in various oncogenic mechanisms, which deserve to be elucidated, in order to find out effective therapies.

## 11. Conclusions

**Figure 2:**
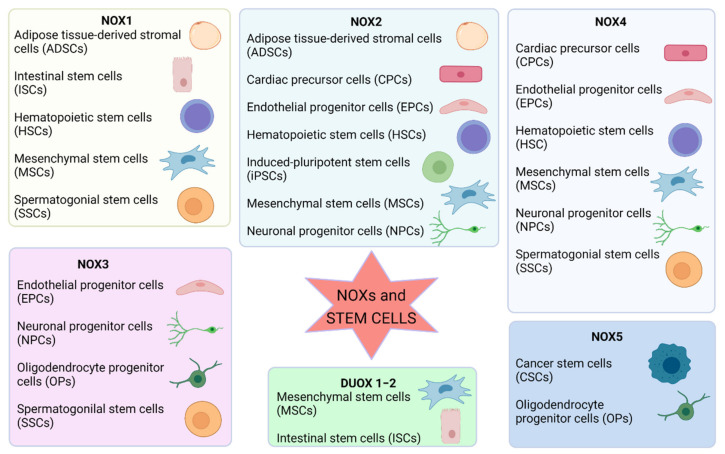
Summary of the involvement of NOX isoforms in different types of stem cells. Image created with BioRender.com (accessed on 1 May 2021).

The importance of ROS in controlling cellular signaling, metabolism, and gene expression is already accepted. Furthermore, the redox state is positively involved in the complex orchestration of stem cell biology, suggesting that NOX-derived ROS are pivotal in the redox cellular signaling in the stem niche (Figure 2).

In general, low ROS levels are functional to maintain stemness, but their increase triggers differentiation, and, depending on the level of ROS, different lineages can be obtained. NOXs are fundamental in this mechanism as they are the main source of ROS that can be finely regulated by the cells. Interestingly, each kind of stem cell possesses a different pattern of NOX isoforms, indicating that they play different functions in the different tissues. For example, NOX4 is the main NOX isoform involved in NSC differentiation, and NOX2 mediates proliferation; mesenchymal cell migration requires NOX4 and DUOX1/2 enzymes, NOX2 mediates progenitor and early endothelial cell proliferation, and NOX4 produces ROS that promote differentiation and stabilization of resulting endothelial cell. ROS produced by NOX1 drive SSC self-renewal, and NOX3 has been demonstrated to be involved in oligodendrocyte differentiation.

However, NOX-derived ROS can also cause dysfunction depending on spatiotemporal NOX expression. Hence, a deeper study of the mechanisms involved in the interplay between NOX and signaling molecules responsible for stem cell fate is imperative if these regulatory mechanisms are to be proposed therapeutically.

Until now, many studies have been carried out on ROS adverse effects in stem and progenitor cells under many pathophysiological conditions, such as hypertension, atherosclerosis, and aging, in which ROS overproduction, generating an oxidative and inflammatory microenvironment, triggers apoptotic mechanisms. On the contrary, a Nox-derived ROS increase exerts positive effects on other processes, such as wound healing, vascular regeneration, and protection from bacterial infection, as reported in Table 1.

Indeed, the modulation of ROS levels has been already exploited as a powerful strategy to improve stem cell transplants; however a better understanding of the molecular mechanisms of how ROS regulate the function of stem and progenitor cells and their niche in physiological and pathological conditions will lead to the development of novel therapeutic strategies, among which the use of NOX specific inhibitors should further help in improving stem cell therapy.

## Figures and Tables

**Table 1:** Nox modulation in stem cells as a potential strategy for the treatment of different pathologies.

Stem Cells	Process	Modulation of Stem Cells	NOX-Derived ROS	References
MSCs	Wound healing	PDGF	↑	[127]
UCB-MSCs	Vascular regeneration	Netrin-1	↑	[128]
BMSCs	Bone commitment	IL-17	↑	[129]
MSCs	Neuronal differentiation	PC-PLC inhibition	↑	[52]
MSCs	Neuroprotection	Apocynin/NOX inhibitor	↓	[130]
BMSCs	Cardioprotection	Acetovanillone	↓	[136]
BMSCs	Aging	Apocynin/NOX inhibitor	↓	[99]
NSCs	Neuroprotection	Angiotensin II	↑	[151,152]
NSCs	Neuro proliferation	chemokine (C-X-C motif) ligand 1	↑	[154]
NSCs	Brain injury protection	NOX2 inhibition	↓	[155]
EPCs	Senescence decrease	Angiotensin II antagonist	↓	[100]
EPCs	Anti-inflammation and neovascularization	Protection from pro-inflammatory cytokines	↑	[159]
EPCs	Antiapoptotic effect	Apocynin/NOX inhibitor	↓	[162]
EPCs	Propapototic effect	Osteoprotegerin	↑	[163]
EPCs	Decrease of EPC function	Hyperlipidemia	↑	[164]
ISCs	Protection from bacteria infection	p-38	↑	[178]
ISCs	Microbiota activation	TLR	↑	[180]
CSCs	Decrease of hepatocellular cancer cell proliferation	PPARγ and Akt inhibitors	↑	[189]
CSCs	Leukemia cell death	dihydrotanshinone	↑	[197]
CSCs	Leukemia cell death	NOX inhibitors and TK inhibitors	↓	[201]

MSCs: Mesenchymal Stem Cells; UCB-MSCs: Umbilical Cord Blood Derived Mesenchymal stem cells; BMSCs Bone-Marrow-derived-Mesenchymal Stem Cells; NSC: Neural Stem Cells; EPCs: Endothelial Progenitor Cells; ISC: Intestinal Stem Cells; CSC Cancer Stem Cells. Arrows describe the increase (↑) or the decrease (↓) of NOX- derived ROS that modulates the biological process.
